# Pyoverdine-induced iron dysregulation exacerbates vascular endothelial barrier dysfunction under hyperglycemic conditions

**DOI:** 10.3389/fendo.2026.1719170

**Published:** 2026-04-17

**Authors:** Guixiang Zhang, Qiyuan Xin, Huimin Zhang, Junxia Yan, Weidong Huang

**Affiliations:** 1 School of Basic Medical Sciences, Ningxia Medical University, Yinchuan, Ningxia, China; 2 People’s Hospital of Ningxia Hui Autonomous Region, Ningxia Medical University, Yinchuan, Ningxia, China

**Keywords:** high glucose, iron homeostasis, mitochondrial function, pseudomonas aeruginosa, pyoverdine

## Abstract

Diabetic foot infection (DFI) remains a leading cause of morbidity and mortality in diabetic patients, with Pseudomonas aeruginosa serving as a predominant pathogen due to its arsenal of virulence factors. Among these, the siderophore pyoverdine (PVD) is critical for bacterial iron acquisition and pathogenesis; however, its direct impact on the vascular endothelial barrier—particularly under hyperglycemic conditions—remains poorly understood. In this study, we employed Human Umbilical Vein Endothelial Cells (HUVECs) as a model to systematically evaluate the effects of PVD and high glucose (HG) on endothelial integrity. Transcriptomic analysis (RNA-seq) revealed that PVD significantly reshaped the gene expression profile of HUVECs, characterized by a marked upregulation of iron homeostasis-related genes, such as TFRC, indicating a state of apparent intracellular iron deficiency. Functional enrichment analysis further highlighted alterations in the extracellular matrix (ECM) and pathways associated with barrier function. *In vitro* assays demonstrated that PVD exposure reduced cell viability, triggered reactive oxygen species (ROS) bursts, and induced a loss of mitochondrial membrane potential. Ultrastructural observations via transmission electron microscopy confirmed pathological changes, including mitochondrial swelling, cristae disorganization, and cytoplasmic vacuolation. Functional assessments showed that PVD significantly increased the transendothelial flux of FITC-dextran and downregulated the expression of the tight junction proteins ZO-1 and Claudin-5, indicating compromised barrier integrity. Notably, these deleterious effects—including oxidative stress, mitochondrial damage, and barrier dysfunction—were significantly exacerbated under HG conditions. Collectively, our findings suggest that PVD impairs the vascular endothelial barrier by disrupting eukaryotic iron homeostasis and activating oxidative stress, leading to the downregulation of junctional proteins. This study identifies PVD as a key mediator of vascular injury in DFI and provides a theoretical rationale for targeting iron metabolism and oxidative stress as a therapeutic strategy.

## 1 Introduction

Diabetes mellitus (DM) is one of the greatest threats to global public health (1). Among its complications, diabetic foot infection is one of the most common in patients with diabetes (2), and the complexity of its pathogenesis poses substantial challenges for effective treatment. *Pseudomonas aeruginosa*, a member of the *Pseudomonas* genus, is an opportunistic pathogen characterized by strong colonization ability, high mutation rates, and multidrug resistance (3–6). In the context of diabetes, host susceptibility to such opportunistic pathogens is increased, resulting in poor wound healing, greater therapeutic challenges, and even progression to amputation in patients with diabetic foot infection (7). The impaired healing of diabetic foot ulcers is largely attributable to host factors; specifically, hyperglycemia damages the function of vascular endothelial cells, and endothelial dysfunction hinders the formation of mature blood vessels, thereby limiting extracellular matrix remodeling, granulation tissue formation, and overall wound repair (8).From the perspective of pathogenic bacteria, *P. aeruginosa* produces a wide array of virulence factors, including siderophores, rhamnolipids, elastase, exotoxin A, exoenzyme S, adhesins, and phospholipase C, all of which contribute to both chronic and acute infections (4, 5, 9). Persistent inflammation caused by these factors prevents wounds from progressing into the proliferative phase, during which angiogenesis is normally promoted, thereby further impairing tissue repair (10, 11). In summary, the effects of bacterial virulence factors on endothelial cells play a pivotal role in the pathogenesis of impaired wound healing (12, 13).

Pyoverdine (PVD), the primary siderophores of *P. aeruginosa*, are considered major virulence factors due to their extremely high affinity for ferric iron. PVD can efficiently sequester iron from host iron-binding proteins, such as transferrin and lactoferrin, thereby providing the bacterium with essential iron resources for growth. In addition, by modulating iron uptake, PVD influence the establishment and maturation of biofilms, and thus are regarded as hallmarks of *P. aeruginosa* virulence (14). Beyond iron acquisition, PVD also regulate the expression of multiple virulence-associated genes through iron homeostasis, indirectly enhancing bacterial pathogenicity. Previous studies have shown that PVD can withdraw iron from host proteins, thereby disturbing host iron metabolic balance, disrupting both iron and mitochondrial homeostasis (15, 16), impairing immune responses, and promoting chronic inflammation and tissue damage. Remarkably, it has also been demonstrated that PVD exert toxic effects on the model organism *Caenorhabditis elegans* even in the absence of bacterial cells (17).However, current therapeutic strategies for diabetes-associated infections primarily focus on glycemic control and conventional antimicrobial approaches, with limited understanding of the cross-disciplinary injury mechanism linking “pathogen iron acquisition–host iron homeostasis.” This knowledge gap restricts the development of mechanism-driven interventions. In the present study, we established an *in vitro* endothelial cell culture model to investigate the effects of the *P. aeruginosa* siderophore pyoverdine (PVD) on vascular endothelial cells. Elucidating the key molecular nodes of PVD-induced endothelial injury under hyperglycemic conditions may provide translational insights and facilitate the design of novel combinational therapeutic strategies for clinical application. Taken together, these considerations highlight the need to explore how PVD disrupts endothelial homeostasis in the diabetic milieu, thereby advancing our understanding of diabetic foot infection pathogenesis and guiding the development of targeted therapeutic interventions.

## 2 Materials and methods

### 2.1 Preparation of PVD

PVD was purchased from Sigma-Aldrich (St. Louis, MO, USA; Cat. No. P8124-1MG). The compound was originally derived from *Pseudomonas fluorescens*. The purity of the PVD powder (1 mg) was verified by high-performance liquid chromatography (HPLC) and was confirmed to be greater than 90%. For stock preparation, PVD powder was dissolved in sterile deionized water to a final concentration of 1 mg/mL, followed by sterile filtration through a 0.22µm membrane filter. The filtered stock solution was aliquoted and stored until use. For experimental treatment, the stock solution was diluted with the complete HUVEC culture medium to the desired working concentrations, while the control groups received an equivalent volume of sterile deionized water.

### 2.2 Cell culture

Human umbilical vein endothelial cells (HUVECs) were obtained from Abcell (Guangzhou, China; Cat. No. AC168). Cells were cultured in the complete HUVEC growth medium provided by the manufacturer (Abcell, Cat. No. ACL168-01), which contains all essential nutrients and growth factors required for endothelial cell proliferation and does not require additional serum or supplements. Cultures were maintained in a humidified incubator at 37 °C with 5% CO_2_. When cell confluence reached 80–90%, cells were dissociated using 0.05% trypsin–EDTA solution (Beyotime, Cat. No. C0202-100) and passaged at a ratio of 1:2–1:3. Cells between passages 3 and 10 were used for experiments.

### 2.3 Cryopreservation and recovery of HUVECs

When HUVECs reached 80–90% confluence and exhibited good morphology, cells were harvested for cryopreservation. After collection, cells were resuspended in pre-cooled freezing medium consisting of 90% fetal bovine serum (FBS; Gibco, USA) and 10% dimethyl sulfoxide (DMSO; Gibco, USA) at a density of 1 × 10^6^ cells/mL. Aliquots were dispensed into sterile cryogenic vials and placed in a controlled-rate freezing container at –80 °C overnight, followed by transfer to liquid nitrogen for long-term storage.

For recovery, frozen vials were rapidly thawed in a 37 °C water bath until only a small ice crystal remained. The cell suspension was then immediately transferred into complete HUVEC culture medium (Abcell, Guangzhou, China; Cat. No. ACL168-01) and centrifuged at 1000 rpm for 5 min to remove residual DMSO. The pellet was resuspended in fresh medium and seeded into culture flasks, followed by incubation at 37 °C in a humidified atmosphere containing 5% CO_2_.

### 2.4 Cell viability assay (CCK-8)

Cell viability was assessed using a Cell Counting Kit-8 (CCK-8; Seven, Beijing, China; Cat. No. SC119). After treatment, the medium was removed and cells were gently rinsed once with serum-free medium. Then 100 μL of complete medium containing 10% CCK-8 reagent (90 μL medium + 10 μL CCK-8) was added to each well and incubated at 37 °C, 5% CO_2_ for 2 h in the dark. OD was read at 450 nm.Cell viability(%)=(ODtest−ODblank)/(ODcontrol−ODblank)×100%,where blank wells contained only medium plus CCK-8 without cells. To determine the working glucose concentration, cells were exposed to graded glucose levels with an osmotic control of equimolar D-mannitol (Solaibio, Beijing, China; Cat. No. 10010518). CCK-8 results were used to select the optimal glucose concentration for subsequent experiments. The same approach was used to determine the working concentration of pyoverdine (PVD).

### 2.5 Measurement of intracellular ROS

Intracellular reactive oxygen species (ROS) levels were measured using the fluorescent probe 2′,7′-dichlorodihydrofluorescein diacetate (DCFH-DA; KeyGEN Biotech, Nanjing, China; Cat. No. KGT010-1). After treatment, cells were washed twice with phosphate-buffered saline (PBS) and incubated with serum-free medium containing 10 μM DCFH-DA at 37 °C for 20 min. During incubation, plates were gently agitated every 10 min to facilitate probe entry into the cells. Following incubation, cells were thoroughly washed three times with PBS to remove excess probe. Fluorescence was observed using a fluorescence microscope (Ex/Em = 488/525 nm), and the fluorescence intensity was quantified using ImageJ software.

### 2.6 Assessment of mitochondrial membrane potential

Mitochondrial membrane potential was evaluated using the JC-1 assay kit (KeyGEN Biotech, Nanjing, China; Cat. No. KGA601). After treatment, cells were washed twice with PBS and incubated with JC-1 working solution at 37 °C for 20 min. Following incubation, the dye solution was removed, and cells were washed twice with PBS. Images were captured under a fluorescence microscope to detect red (JC-1 aggregates) and green (JC-1 monomers) signals. The ratio of red to green fluorescence was calculated using ImageJ software to assess changes in ΔΨm. A decreased red/green fluorescence ratio was interpreted as mitochondrial depolarization, indicative of mitochondrial dysfunction.

### 2.7 Transmission electron microscopy

After the indicated treatments, HUVECs were washed three times with buffer and fixed in 2.5% glutaraldehyde at 4 °C. After washing, cells were post-fixed in 1% osmium tetroxide, followed by graded ethanol dehydration, resin infiltration, and embedding. Ultrathin sections were prepared using an ultramicrotome, mounted on copper grids, and stained with uranyl acetate and lead citrate. Ultrastructural changes were observed and imaged using a transmission electron microscope (TEM) at magnifications of 10,000× and 30,000×.

### 2.8 HUVEC transwell permeability assay

Transwell inserts (0.4 μm pore size, 24-well format; Corning) were used to assess endothelial monolayer permeability. HUVECs were seeded in the upper chamber at 1 × 10^5^ cells/insert in 200 μL medium, while 600 μL complete medium was added to the lower chamber. Cells were cultured for 24 h at 37 °C with 5% CO_2_ to allow formation of a confluent monolayer, followed by 24 h of treatment according to the experimental groups. After treatment, the medium was removed and cells were gently washed with PBS. The lower chamber was filled with 600 μL phenol red–free medium, and 200 μL FITC-dextran (4 kDa) solution was added to the upper chamber. Following 60 min incubation at 37 °C in the dark, 100 μL medium from the lower chamber was collected and transferred to a black 96-well plate. Fluorescence intensity was measured using a microplate reader (Ex/Em = 485/530 nm). FITC-dextran concentrations were calculated using a standard curve and used for statistical analysis.

### 2.9 RNA sequencing

RNA sequencing was performed by Beijing Genomics Bioservices Co., Ltd. (Beijing, China). RNA quality was assessed by measuring concentration with a Qubit 4.0 Fluorometer (Thermo Fisher Scientific) and evaluating fragment size and integrity with a Qsep400 system (BiOptic, Taiwan). Qualified RNA samples were used to construct libraries with the MGIEasy RNA Library Prep Kit (MGI Tech, Shenzhen, China). Briefly, poly(A)+ mRNA was enriched using Oligo(dT)-attached magnetic beads, fragmented with Fragmentation Buffer, and reverse-transcribed into double-stranded cDNA using random hexamer primers, followed by purification. The cDNA was then subjected to end repair, A-tailing, adaptor ligation, and PCR amplification. After quality control, amplified products were circularized and converted into DNA nanoballs (DNBs) via rolling circle amplification (RCA), which were subsequently loaded onto sequencing chips. Finally, sequencing was carried out on the DNBSEQ-T7 platform (MGI Tech, Shenzhen, China) to generate 150 bp paired-end reads.

### 2.10 RNA extraction and quantitative real-time PCR

HUVECs were cultured under standard conditions for 24 h and subsequently treated with the indicated media according to experimental groups. Total RNA was extracted from each group using the Total RNA Extraction Kit (UElandy Biotech, Suzhou, China; Cat. No. UE-MN-MS-RNA-50), following the manufacturer’s instructions. RNA concentration and purity were determined with a NanoDrop 2000 spectrophotometer (Thermo Fisher Scientific, USA), and samples with an A260/A280 ratio between 1.8 and 2.0 were used for downstream experiments.Complementary DNA (cDNA) was synthesized from total RNA using a reverse transcription kit (TransGen Biotech, Beijing, China; Cat. No. AU341). qRT-PCR was performed using gene-specific primers and DNA polymerase to amplify the target genes. The primer sequences used in this study are listed in **Table 1**. β-ACTIN was used as the internal control for normalization. Relative mRNA expression levels were calculated using the 2^−ΔΔCt method. All experiments were conducted in triplicate.

**Table 1:** Primer sequences used for qRT-PCR in this study.

Gene	Forward primer (5′–3′)	Reverse primer (3′–5′)
STC1	cca gcc tct tcc aca tcc tg	ctc att ggt gcg tct cct gt
SDC1	ggc tat tcc cac gtc tcc ag	gac tac agc ctc tcc ctc ct
SULF1	agg agg ctg ctc agg aag ta	cca gtg gtt gtt gtc atg cg
GPX1	latg tgt gct gct cgg cta g	agt acc ttg ccc cgc ag
FDXR	gga gct gga gcc aga cct gag	ctg cct tcg tga tgt ccg ttc tc
ANGPTL4	cga tgg ctc agt gga ctt caa cc	ccg tga tgc tat gca cct tct cc
GCLM	cgc aca gcg agg agc ttc atg	aac tcc ctg acc aaa tct ggg ttg
TFRC	tga ggg agg agc cag gag agg	ctt gat ggt gcc ggt gaa gtc tg
β-ACTIN	cct ggc acc cag cac aat	ggg ccg gac tcg tca tac

(All primers were synthesized by Sangon Biotech, Shanghai, China).

### 2.11 Western blot analysis

Proteins were extracted from HUVECs using RIPA buffer (Servicebio, Wuhan, China) supplemented with protease and phosphatase inhibitors, and quantified using a BCA protein assay kit (Seven, Beijing, China). Equal amounts of protein (20–30 μg) were separated on 4–20% precast gradient SDS–PAGE gels (Seven, Beijing, China) and transferred onto PVDF membranes (Millipore, USA). Membranes were blocked with 5% non-fat dry milk for 1 h at room temperature and incubated overnight at 4 °C with primary antibodies against ZO-1 (1:2000; Sanying Biotech, Wuhan, China) and Claudin-5 (1:2000; Sanying Biotech, Wuhan, China). GAPDH (1:5000; ab8245, Abcam, UK) was used as the loading control. After washing, membranes were incubated with fluorescently labeled secondary antibodies (1:5000; LI-COR Biosciences, USA) for 1 h at room temperature. Fluorescent signals were detected using an Odyssey CLx imaging system (LI-COR Biosciences) and quantified with ImageJ software (NIH, USA).

### 2.12 Statistical analysis

Statistical analyses were performed using GraphPad Prism 6 (GraphPad Software, San Diego, CA, USA). All data are presented as mean ± standard deviation (SD) from at least three independent experiments. For comparisons between two groups, the unpaired Student’s t-test was employed. For comparisons among multiple groups, one-way analysis of variance (ANOVA) followed by Tukey’s *post hoc* test was used. Statistical significance was defined as *P* < 0.05.

## 3 Results

### 3.1 Effects of high glucose and PVD on the viability of HUVECs

**Figure 1:**
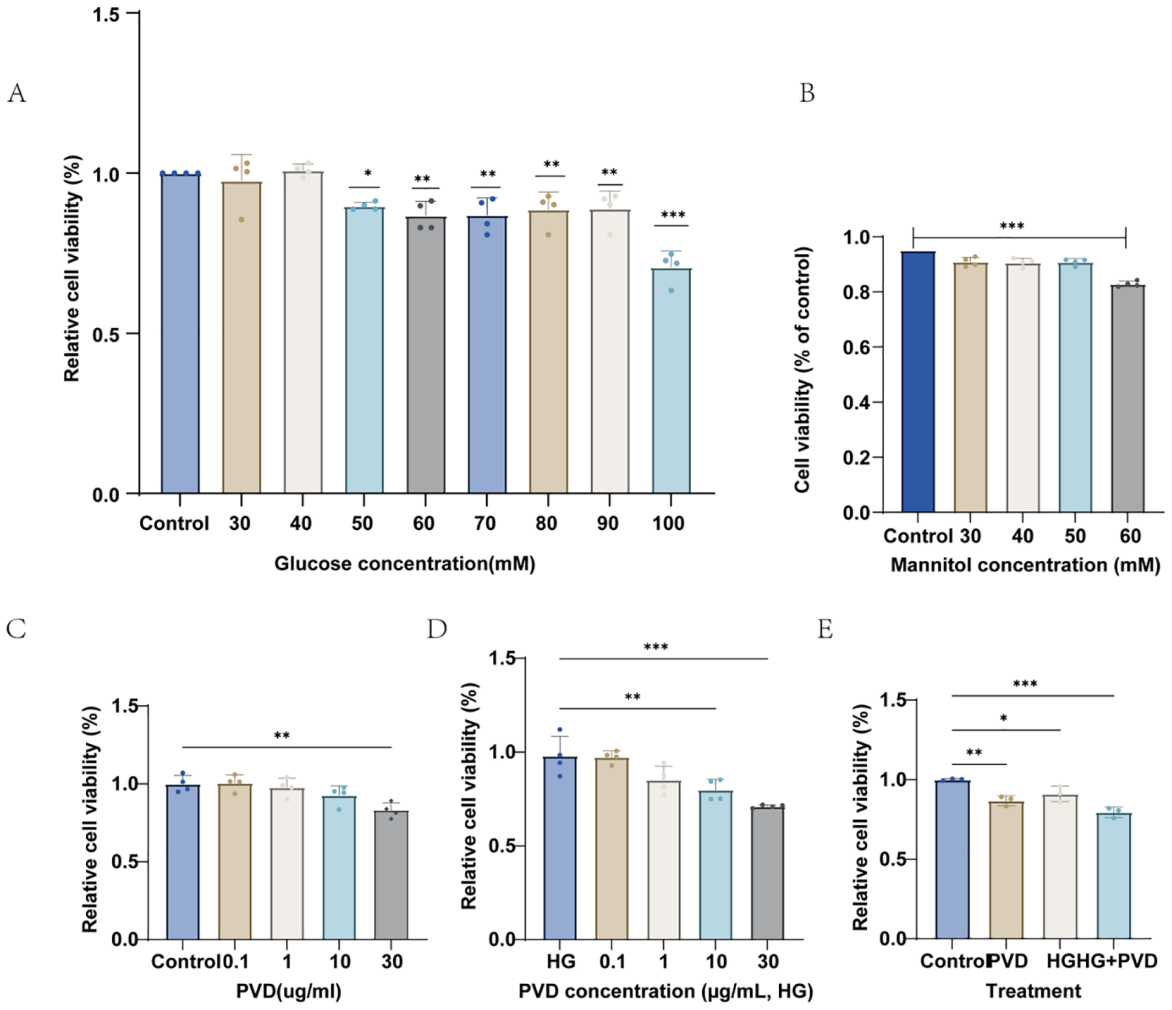
Effects of high glucose and pyoverdine (PVD) on HUVEC viability. HUVEC viability was assessed by CCK-8 assay under different experimental conditions. **(A)** Cells were exposed to increasing concentrations of glucose (30–100 mM). **(B)** Mannitol (30–60 mM) was used as an osmotic control to evaluate the effect of hyperosmolarity. **(C)** Cells were treated with different concentrations of PVD (0.1–30 μg/mL). **(D)** Cells were treated with PVD (0.1–30 μg/mL) under high-glucose (HG) conditions. **(E)** Comparison of cell viability among Control, PVD, HG, and HG + PVD groups. Data are presented as mean ± SD and normalized to the control group. ^*^P < 0.05, ^**^P < 0.01, ^***^P < 0.001 vs. Control.

To determine the appropriate concentration of high glucose for subsequent experiments, HUVECs were treated with glucose ranging from 30 to 100 mM for 24 h, and cell viability was measured using the CCK-8 assay. As shown in **Figure 1A**, cell viability gradually decreased with increasing glucose concentration. A significant reduction was observed at 50 mM compared with the normal control (5.5 mM), and a further decline occurred at higher concentrations.To exclude the potential effect of osmotic stress, HUVECs were treated with equimolar concentrations of mannitol (30–60 mM). Mannitol had no significant effect on cell viability at concentrations below 60 mM, whereas a reduction in viability was observed at 60 mM (**Figure 1B**), suggesting that osmotic stress had minimal influence within the experimental range. Therefore, 50 mM glucose was selected as the high-glucose condition for subsequent experiments.Next, the effect of PVD on HUVEC viability was evaluated. Under normal glucose conditions (5.5 mM), PVD had little effect on cell viability at concentrations up to 10 μg/mL, whereas a significant decrease was observed at 30 μg/mL (**Figure 1C**). Under high-glucose conditions (50 mM), PVD further reduced cell viability, and the inhibitory effect became more pronounced with increasing concentrations (**Figure 1D**). Furthermore, comparison of the treatment groups showed that high glucose significantly reduced cell viability compared with the control group, while PVD alone had only a mild effect. Notably, combined treatment with high glucose and PVD resulted in a greater reduction in cell viability (**Figure 1E**). Based on these results, 30 μg/mL PVD was selected for subsequent experiments.

### 3.2 PVD Aggravates oxidative stress and induces mitochondrial dysfunction in HUVECs under high-glucose conditions

**Figure 2:**
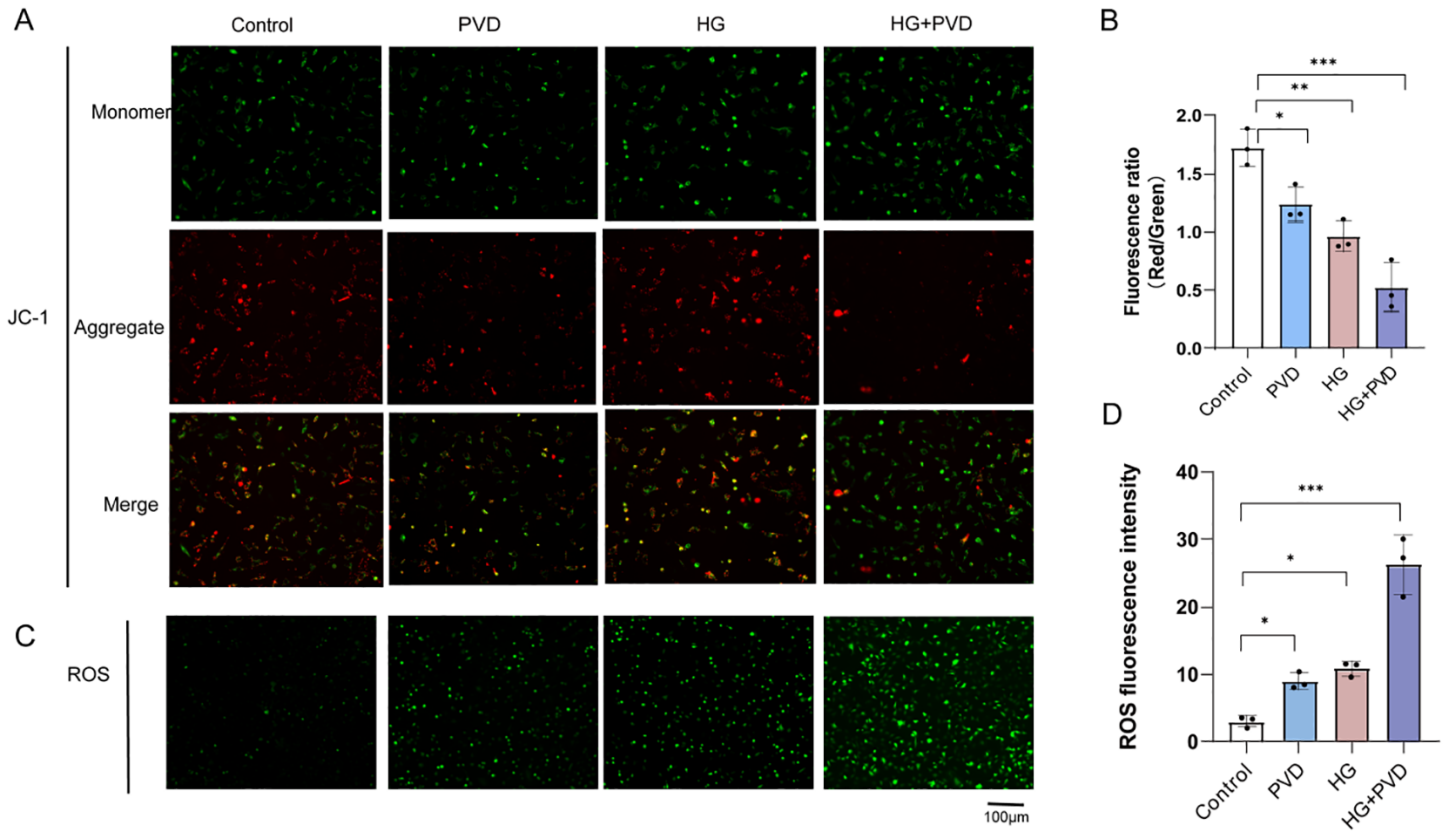
Effects of PVD and high glucose on mitochondrial membrane potential and ROS production in HUVECs. **(A)**. Representative fluorescence images of JC-1 staining in HUVECs under different treatments. Green fluorescence indicates JC-1 monomers, whereas red fluorescence indicates JC-1 aggregates. **(B)**. Quantification of mitochondrial membrane potential expressed as the red/green fluorescence ratio. **(C)**. Representative fluorescence images of intracellular ROS levels detected by DCFH-DA staining. **(D)**. Quantitative analysis of ROS fluorescence intensity.Scale bar = 100 μm. Data are presented as mean ± SD (n = 3). ^*^P < 0.05, ^**^P < 0.01, ^***^P < 0.001, vs. Control.

To investigate whether PVD enhances oxidative stress and mitochondrial dysfunction in endothelial cells under high-glucose (HG) conditions, intracellular ROS levels and mitochondrial membrane potential (ΔΨm) were evaluated using DCFH-DA and JC-1 staining, respectively. As shown in **Figures 2A, B**, JC-1 staining revealed that both PVD and HG treatment alone reduced mitochondrial membrane potential compared with the control group, as indicated by a decreased red/green fluorescence ratio. Notably, the HG + PVD group exhibited the most pronounced reduction in ΔΨm, suggesting aggravated mitochondrial depolarization and more severe mitochondrial dysfunction.Consistently, ROS staining demonstrated that intracellular ROS levels were increased in both the PVD and HG groups relative to the control group (**Figures 2C, D**). Importantly, the HG + PVD group showed the highest ROS production among all groups, indicating that PVD further enhances HG-induced oxidative stress.

Together, these findings indicate that PVD aggravates oxidative stress and mitochondrial dysfunction in HUVECs under high-glucose conditions.

### 3.3 Transmission electron microscopy analysis of ultrastructural changes in HUVECs

**Figure 3:**
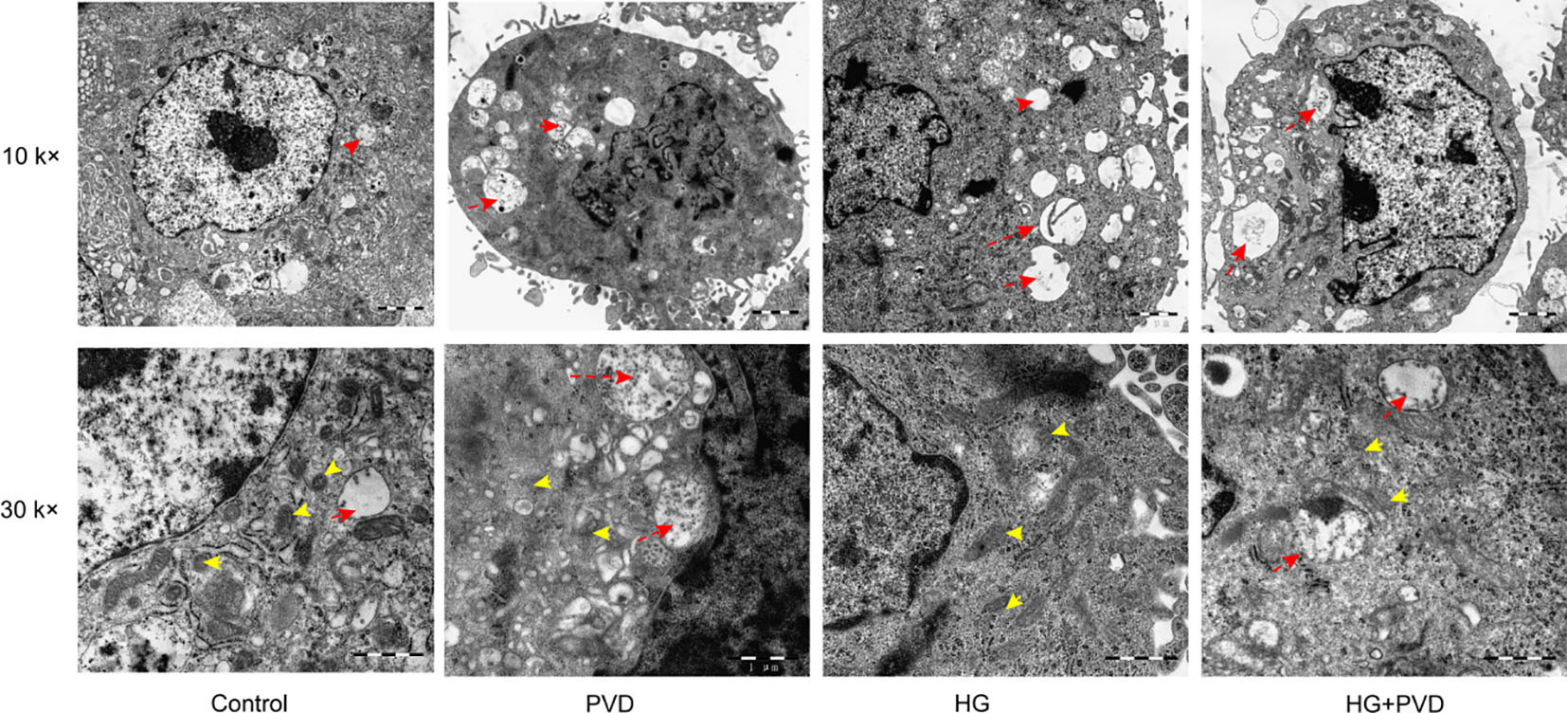
Ultrastructural changes of HUVECs under different treatment conditions observed by transmission electron microscopy (TEM).The ultrastructure of HUVECs under different treatment conditions was examined by TEM. The upper panels show images at 10,000× magnification, illustrating overall cellular morphology, whereas the lower panels show images at 30,000× magnification, highlighting mitochondrial ultrastructure. Yellow arrows indicate mitochondria, and red arrows indicate cytoplasmic vacuole-like structures. Compared with the control group, cells in the PVD and HG groups exhibited obvious mitochondrial abnormalities, including mitochondrial swelling, reduced matrix electron density, and disorganization of cristae, accompanied by the formation of cytoplasmic vacuole-like structures. These alterations were further aggravated in the HG + PVD group, characterized by pronounced mitochondrial swelling, severe disruption or loss of cristae, and a marked increase in vacuole-like structures.

To further evaluate the effects of different treatments on endothelial cell ultrastructure, HUVECs were examined by transmission electron microscopy (**Figure 3**). In the Control group, cells exhibited intact structures with regular nuclear morphology and evenly distributed cytoplasmic organelles. Mitochondria appeared oval or short rod-shaped, with intact double membranes, well-organized and dense cristae, and homogeneous matrix electron density, without obvious structural abnormalities. In the PVD-treated group, some mitochondria showed varying degrees of swelling, reduced matrix electron density, and disorganized or partially disrupted cristae. In addition, multiple vacuole-like structures were observed in the cytoplasm, suggesting stress-related intracellular vesicular alterations. In the HG group, mitochondrial abnormalities were also observed, including mild mitochondrial swelling and blurred cristae structures, accompanied by the presence of a small number of cytoplasmic vacuole-like structures, indicating that high-glucose conditions induced a certain degree of ultrastructural damage in endothelial cells. In the HG + PVD group, cellular damage was more pronounced. A large number of mitochondria exhibited marked swelling, with severely disrupted or partially lost cristae and further decreased matrix electron density. Meanwhile, vacuole-like structures in the cytoplasm were significantly increased, with some appearing as large vesicle-like structures, indicating substantial disruption of intracellular membrane systems and organelle homeostasis. Overall, PVD treatment induced mitochondrial ultrastructural damage in HUVECs accompanied by the formation of vacuole-like structures, and this damage was further aggravated under high-glucose conditions.

### 3.4 PVD treatment induces widespread transcriptional changes in endothelial cells

**Figure 4:**
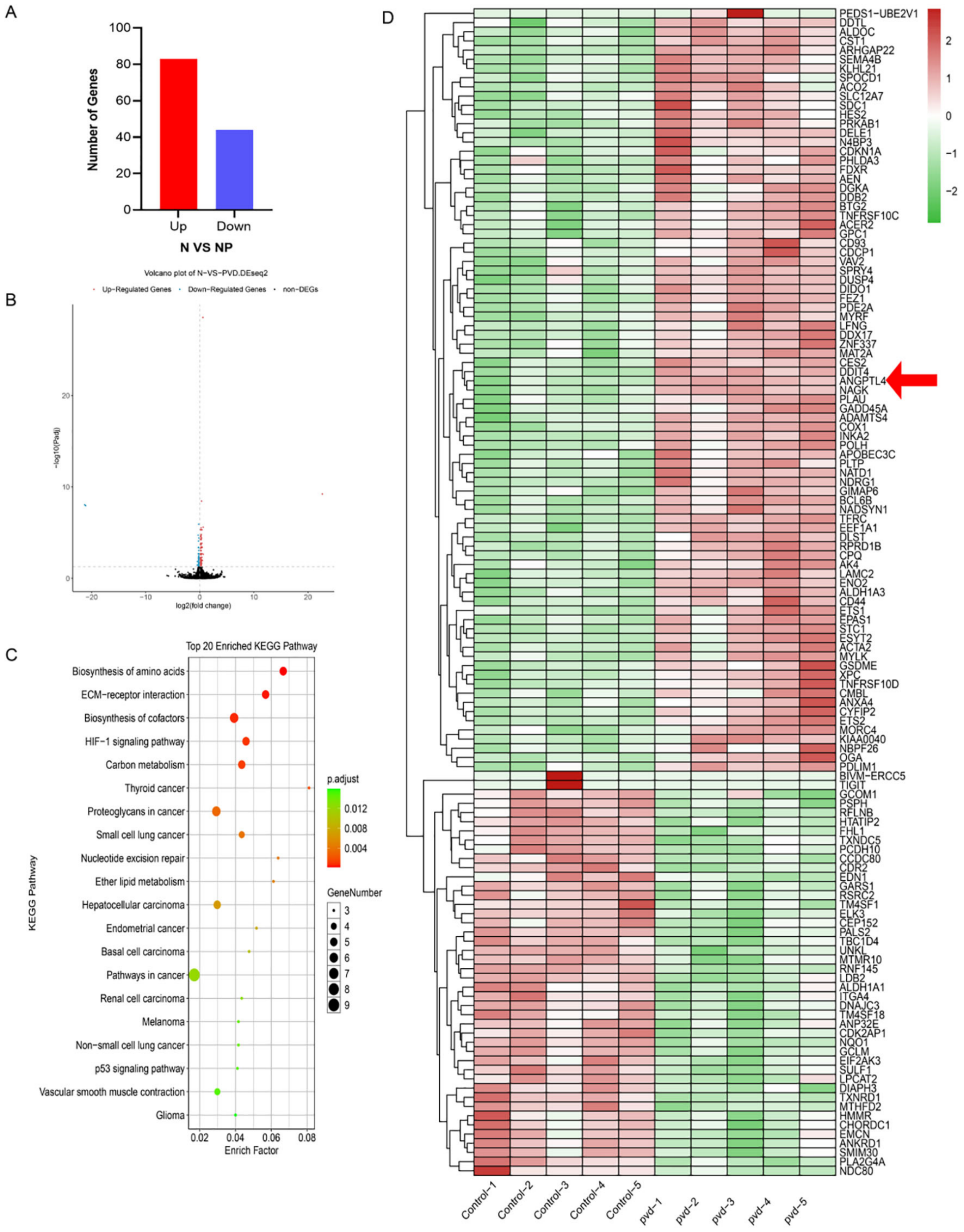
Transcriptomic analysis of HUVECs in response to PVD treatment. **(A)** Bar plot showing the numbers of upregulated and downregulated differentially expressed genes (DEGs). **(B)** Volcano plot of DEGs. The x-axis represents log_2_(fold change) and the y-axis represents −log_10_(P value). Red dots indicate upregulated genes, blue dots indicate downregulated genes, and black dots indicate non-significant genes. **(C)** KEGG pathway enrichment analysis of DEGs. The x-axis represents the enrichment factor; bubble size indicates the number of enriched genes, and color represents the adjusted P value (padjust). **(D)** Hierarchical clustering heatmap of differentially expressed genes. Each column represents a sample and each row represents a gene. Red indicates relatively higher expression, whereas green indicates relatively lower expression.

To further investigate the impact of PVD on endothelial gene expression, RNA sequencing was performed to compare the control and PVD-treated groups. Based on the criteria for differential gene expression (|log_2_FC| ≥ 1, FDR < 0.05), a total of 127 differentially expressed genes (DEGs) were identified, including 83 upregulated and 44 downregulated genes (**Figure 4A**). The overall distribution of DEGs was further illustrated by a volcano plot (**Figure 4B**). Hierarchical clustering analysis demonstrated distinct expression patterns between the control and PVD-treated groups, clearly separating the two groups in the heatmap (**Figure 4D**). Notably, ANGPTL4 was markedly upregulated following PVD treatment.Subsequent KEGG pathway enrichment analysis of the DEGs revealed significant enrichment in pathways related to biosynthesis of amino acids, ECM–receptor interaction, HIF-1 signaling pathway, carbon metabolism, proteoglycans in cancer, and the p53 signaling pathway (**Figure 4C**). These findings suggest that PVD may contribute to endothelial dysfunction through multiple mechanisms, particularly involving metabolic regulation, extracellular matrix remodeling, and oxidative stress–related signaling pathways.

### 3.5 Identification and qPCR confirmation of DEGs associated with endothelial dysfunction pathways

**Figure 5:**
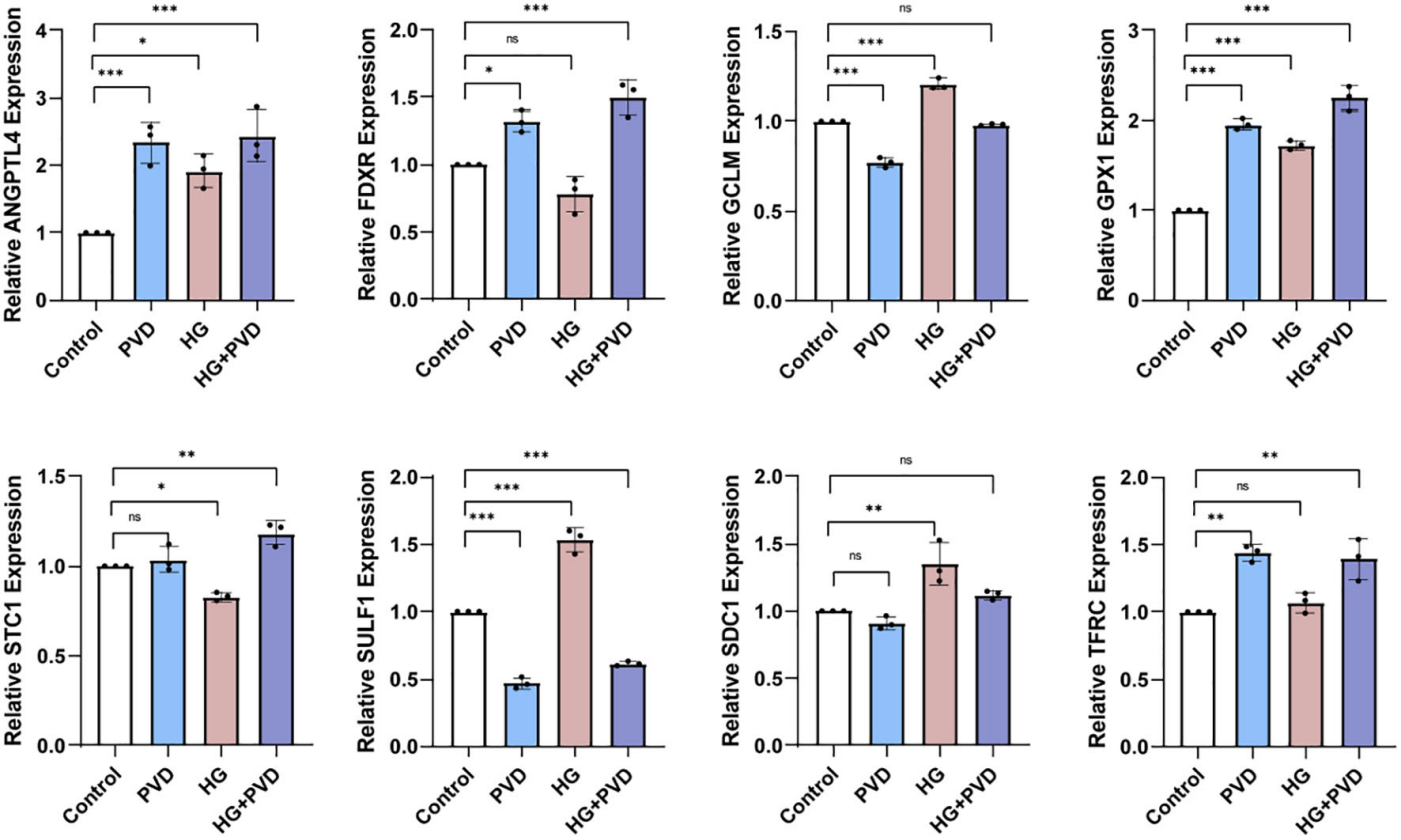
Validation of selected differentially expressed genes by qPCR. Relative mRNA expression levels of ANGPTL4, FDXR, GCLM, GPX1, STC1, SULF1, SDC1, and TFRC in HUVECs under different treatment conditions. Data are representative of three independent experiments (n = 3) and are presented as mean ± SD. Statistical analyses were performed as described in Materials and Methods. ^*^P < 0.05, ^**^P < 0.01, ^***^P < 0.001 vs. control; ns, not significant.

Based on the transcriptomic results, and considering both the magnitude of expression changes and KEGG enrichment, we further selected eight DEGs related to iron homeostasis, oxidative stress, and extracellular matrix remodeling for validation by qPCR (**Figure 5**).

Transcriptomic analysis revealed that the iron metabolism–related gene TFRC showed no significant change in the HG group, whereas it was significantly upregulated in both the PVD and HG + PVD groups (*P* < 0.01). These findings suggest that exogenous PVD can modulate iron homeostasis in endothelial cells by altering the transcription of iron metabolism–related genes, and this effect remains evident under hyperglycemic conditions.

In the context of oxidative stress and antioxidant defense, the expression of GPX1, FDXR, and STC1 was differentially regulated. GPX1 was markedly upregulated in the PVD, HG, and HG + PVD groups (*P* < 0.001), indicating activation of glutathione metabolism and peroxide detoxification pathways under both stimuli. FDXR expression remained unchanged under HG treatment but was significantly increased following PVD exposure, suggesting that PVD alone can initiate oxidative stress–related responses. STC1 showed no significant change with HG treatment alone but was upregulated in the HG + PVD group, indicating that PVD more effectively triggers its expression under hyperglycemic conditions. In contrast, GCLM, a gene implicated in both antioxidant defense and iron metabolism, exhibited no significant change under HG conditions but showed a downward trend after PVD treatment, implying a potential dual regulatory effect of PVD on oxidative stress and iron homeostasis pathways.

In terms of metabolic regulation and extracellular matrix (ECM) remodeling, ANGPTL4, SULF1, and SDC1 were all upregulated following HG treatment. Among them, ANGPTL4 was significantly increased in the PVD, HG, and HG + PVD groups, with the highest expression observed in the HG + PVD group (*P* < 0.001), indicating a pronounced synergistic effect. SDC1 was upregulated by both PVD and HG treatments (*P* < 0.01), whereas the combined HG + PVD treatment did not elicit a further synergistic increase (*P* > 0.05). In contrast, SULF1 expression was significantly elevated under HG conditions but markedly decreased in the PVD group (*P* < 0.001), and remained suppressed in the HG + PVD group, suggesting that PVD exerts a dominant regulatory effect on this gene. Taken together, these findings indicate that PVD may influence endothelial cell function by modulating the expression of ECM-related genes.

Overall, the qPCR validation results were largely consistent with the transcriptomic data, suggesting that PVD influences endothelial cell function by regulating multiple signaling pathways, including oxidative stress, iron homeostasis, and extracellular matrix remodeling. Based on these findings, subsequent experiments will evaluate the effect of PVD on endothelial permeability at the protein level.

### 3.6 PVD downregulates tight junction proteins associated with endothelial barrier integrity

**Figure 6:**
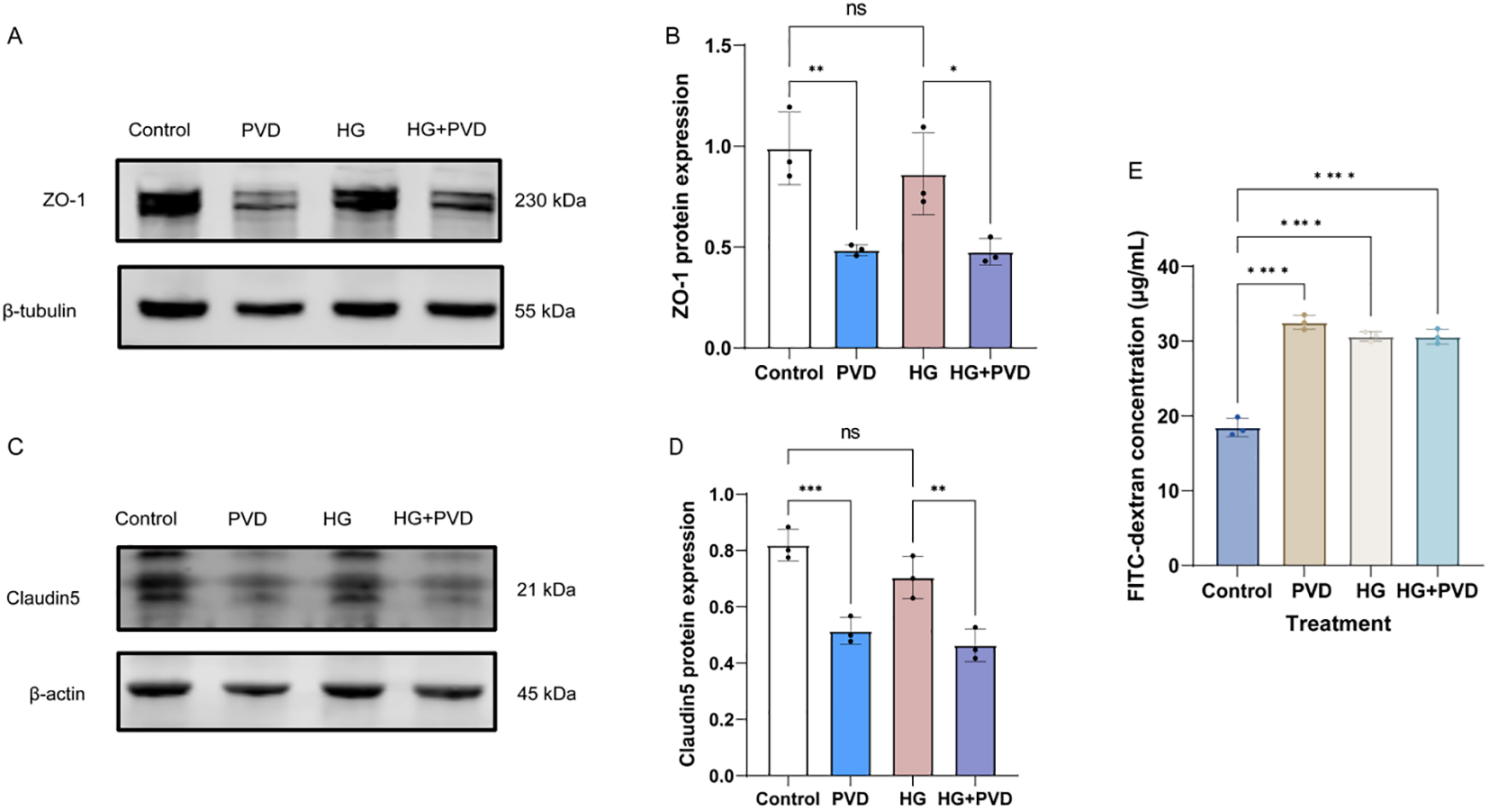
Effects of PVD and high glucose on tight junction protein expression and endothelial permeability in HUVECs. **(A)** Representative Western blot images of ZO-1 expression in HUVECs under different treatments. β-tubulin was used as the loading control. **(B)** Quantification of ZO-1 protein expression. **(C)** Representative Western blot images of Claudin-5 expression. β-actin was used as the loading control. **(D)** Quantification of Claudin-5 protein expression. **(E)** Endothelial permeability assessed by FITC-dextran (4 kDa) Transwell assay.Data are presented as mean ± SD (n = 3). ^*^P < 0.05, ^**^P < 0.01, ^***^P < 0.001,^****^P < 0.0001. ns, not significant. PVD, pyoverdine.

To investigate the effects of PVD and high glucose on endothelial barrier integrity, the expression levels of tight junction proteins ZO-1 and Claudin-5 were analyzed by Western blot. As shown in **Figures 6A, B**, PVD treatment significantly decreased ZO-1 protein expression compared with the control group (*P* < 0.01), whereas HG alone did not show a significant change. Similarly, analysis of Claudin-5 expression revealed a significant reduction in the PVD group compared with the control group (*P* < 0.001), while HG treatment alone showed no significant effect (**Figures 6C, D**). To further assess endothelial barrier function, permeability was evaluated using a FITC-dextran Transwell assay. As shown in **Figure 6E**, both PVD and HG treatments significantly increased FITC-dextran flux compared with the control group (*P* < 0.001). Notably, the HG + PVD group exhibited the highest FITC-dextran permeability, indicating that the combined treatment further impaired endothelial barrier integrity.

## 4 Discussion

Diabetes is a metabolic disorder characterized by chronic hyperglycemia, which impairs host immunity and induces vascular endothelial dysfunction, thereby increasing both the incidence and severity of bacterial infections (18–20). Pseudomonas aeruginosa is a pathogen with opportunistic characteristics that readily infects patients with diabetes. Epidemiological data indicate that P. aeruginosa has become the most common Gram-negative pathogen isolated from diabetic foot infections (21) The pathogenicity of P. aeruginosa is complex, as it secretes a variety of virulence factors. Among these, siderophores, which participate in iron acquisition, transport, and homeostasis, are considered one of its major virulence determinants (22) Pyoverdine (PVD), a well-known siderophore of P. aeruginosa, has been reported to disrupt mitochondrial homeostasis and induce mitophagy in Caenorhabditis elegans (15, 16). Kang et al. further demonstrated in C. elegans models that pyoverdine can modulate host immune responses (22–25), highlighting its importance in host–pathogen interactions (26, 27) Endothelial cells are the principal components of blood vessels. They rest on the basement membrane at their basolateral side and face the bloodstream at their apical/luminal side. In most organs, endothelial cells form a dynamic barrier between the blood and the surrounding tissues. In endothelial cell infection models, the type III secretion system (T3SS) and the elastase LasB of P. aeruginosa have been shown to disrupt endothelial junctional integrity and permeability, which represents a key mechanism facilitating bacterial dissemination within the host (3, 28) Current evidence regarding the impact of PVD on vascular endothelial function and its underlying molecular mechanisms remains insufficient. In particular, it is unclear how PVD affects endothelial cell function under hyperglycemic conditions. Therefore, this study was designed to investigate the effects of PVD on endothelial cells *in vitro* and to elucidate the potential mechanisms involved.

Regarding the effects of high glucose on endothelial cell viability, most studies have indicated that the addition of 30 mM glucose to the culture medium is generally considered the standard diabetic-like condition (29, 30). However, many reports have also employed 50 mM glucose to establish diabetic models (31, 32). In the present study, we established a glucose concentration gradient and performed CCK-8 assays. As shown in **Figure 1A**, exposure to 50 mM glucose for 24 h significantly reduced endothelial cell viability. The mannitol control group demonstrated that at 50 mmol/L no osmotic effect was observed on endothelial cell viability, indicating that the reduction in viability was specifically due to glucose. Therefore, we selected 50 mM glucose to mimic the diabetic environment in subsequent experiments.

To determine the appropriate concentration of pyoverdine (PVD), we treated cells under both normal glucose (N) and high glucose (HG) conditions with a concentration gradient of purified PVD (P, HG+P) and assessed viability using the CCK-8 assay. In the normal glucose group, PVD at 30 µg/mL for 24 h reduced HUVEC viability by approximately 20% compared with the control group, a statistically significant difference (**P<0.01). Under high glucose conditions, PVD at 10 µg/mL for 24 h was sufficient to significantly reduce HUVEC viability (**P<0.01). These results indicate that PVD reduces endothelial cell viability *in vitro*, and that under high glucose conditions, lower PVD concentrations are sufficient to exert cytotoxic effects, suggesting that hyperglycemia may exacerbate PVD-induced endothelial injury.

To our knowledge, no previous studies have examined the effects of PVD on endothelial cell models. However, Kirienko and colleagues reported that PVD can induce hypoxic responses and cell death in the absence of bacteria (17, 33). Their *in vitro* studies demonstrated that pyoverdine can enter human bronchial epithelial cells and also promote the production of another extracellular factor, rhamnolipid, thereby influencing host cell viability. Our findings further confirm that PVD alone can impair cell viability *in vitro* in the absence of bacteria. Nevertheless, the precise molecular mechanisms underlying this effect require further investigation.Oxidative stress refers to an imbalance between oxidation and antioxidation within the organism, leading to excessive accumulation of intracellular free radicals such as reactive oxygen species (ROS), which subsequently cause cellular damage, dysfunction, and even death (34). In patients with diabetes, vascular endothelial cells are commonly subjected to oxidative stress, which disrupts endothelial junctions, increases vascular permeability, and ultimately contributes to the development of both macrovascular and microvascular complications (35). In the present study, ROS levels were elevated and mitochondrial membrane potential (JC-1) was depolarized in the HG group compared with the control group, findings consistent with most previous reports.With respect to the effects of PVD on endothelial cell mitochondrial function, no direct studies have been identified to date. Only a few *in vivo* experiments (15, 33) have demonstrated that PVD directly reduces mitochondrial membrane potential and ATP production in model organisms, thereby causing mitochondrial dysfunction.

Our findings demonstrate that PVD treatment significantly elevates intracellular ROS levels and induces a decline in mitochondrial membrane potential (ΔΨm) compared with the control group, indicating the onset of oxidative stress and mitochondrial dysfunction. Ultrastructural analysis using transmission electron microscopy (TEM) further confirmed mitochondrial injury in the PVD-treated group, characterized by mitochondrial swelling, reduced matrix electron density, and disorganized or locally fragmented cristae. In addition, multiple vacuole-like structures were observed in the cytoplasm, suggesting stress-associated alterations in intracellular vesicular dynamics. Notably, under hyperglycemic conditions, PVD further amplified ROS production and exacerbated mitochondrial depolarization and structural damage in endothelial cells, indicating that hyperglycemia synergistically enhances the deleterious effects of PVD on mitochondrial homeostasis.

To explore the molecular mechanisms underlying PVD-induced mitochondrial dysfunction and endothelial barrier impairment, transcriptomic sequencing (RNA-seq) was performed comparing the Control and PVD groups. Among the differentially expressed genes, TFRC (transferrin receptor) and FDXR (ferredoxin reductase) showed the most prominent upregulation. TFRC mediates cellular iron uptake through transferrin binding and receptor-mediated endocytosis and plays a critical role in maintaining intracellular iron homeostasis. Because TFRC expression is tightly regulated by intracellular labile iron pools and is typically upregulated during iron deficiency, these results suggest that PVD effectively sequesters iron from the extracellular environment, triggering a transcriptional stress response associated with iron deprivation. This observation provides evidence that PVD alone, even in the absence of viable bacteria, can directly modulate the transcriptional landscape of host endothelial cells.

KEGG pathway enrichment analysis further revealed significant enrichment of pathways associated with biosynthesis of amino acids, carbon metabolism, and biosynthesis of cofactors, suggesting metabolic adaptation to PVD-induced stress (36, 37). In addition, enrichment of pathways related to ECM–receptor interaction and proteoglycans in cancer indicates potential remodeling of extracellular matrix components and altered cell–matrix interactions, which may contribute to endothelial barrier instability (38). The enrichment of the HIF-1 signaling pathway further suggests that PVD may induce pseudo-hypoxic signaling or severe oxidative stress, thereby promoting endothelial dysfunction.

Subsequent qPCR validation of key genes confirmed the reliability of the transcriptomic results. The upregulation of antioxidant genes such as GPX1 and GCLM likely reflects a compensatory response to increased oxidative stress. In contrast, the downregulation of SULF1 may weaken extracellular matrix stability, whereas the upregulation of ANGPTL4 is consistent with increased endothelial permeability.

To functionally validate these molecular alterations, endothelial barrier integrity was assessed using FITC-dextran permeability assays and tight junction protein analysis. Both high glucose (HG) and PVD treatments increased endothelial permeability compared with the control group. However, only PVD treatment significantly reduced the expression of the tight junction proteins ZO-1 and Claudin-5, whereas HG treatment alone did not cause a significant decrease in these junctional proteins (P > 0.05). These findings suggest that hyperglycemia may increase endothelial permeability primarily through metabolic stress and cellular dysfunction, while PVD directly disrupts tight junction integrity and barrier structure. Together, these results indicate that PVD is a potent inducer of endothelial barrier damage, and hyperglycemia further amplifies this pathological process.

Despite these findings, several limitations of the present study should be acknowledged. First, this study was primarily conducted using an *in vitro* HUVEC model, and therefore the results may not fully reflect the complex physiological environment *in vivo*. Validation in animal models or clinical samples will be necessary to further confirm the biological relevance of these findings. Because pyoverdine (PVD) is a bacterial siderophore, establishing an appropriate *in vivo* model would require specialized infection or toxin exposure systems, which were not available in the present study.

Second, although the transcriptomic analysis revealed significant alterations in iron metabolism–related genes, including the upregulation of TFRC, direct measurement of intracellular iron levels was not performed. The experimental model in this study primarily reflects a cellular iron-deficient state rather than iron overload. Under such conditions, conventional iron staining methods such as Prussian blue staining are often insufficiently sensitive to detect intracellular iron changes. Preliminary experiments using this method did not yield reliable signals, and therefore these data were not included in the manuscript. Future studies employing more sensitive approaches to quantify intracellular iron dynamics would help further clarify the role of PVD in regulating host iron homeostasis.

Third, mechanistic validation experiments, such as gene knockdown or overexpression of key iron metabolism–related genes, were not performed in the present study. Such experiments would help establish a clearer causal relationship between PVD-induced iron dysregulation and endothelial barrier dysfunction.

Despite these limitations, our findings provide new insights into the potential role of the Pseudomonas aeruginosa siderophore PVD in modulating host endothelial cell function. Given that P. aeruginosa is one of the most common Gram-negative pathogens isolated from diabetic foot infections, understanding how its siderophores influence host endothelial barrier integrity may reveal novel aspects of host–pathogen interactions. Future studies integrating *in vivo* models, molecular intervention strategies, and more precise iron metabolism assays will be essential to further elucidate the mechanisms linking PVD-mediated iron dysregulation to endothelial barrier injury and to explore potential therapeutic strategies targeting iron metabolism or oxidative stress in infection-associated vascular complications.

## 5 Conclusions

In summary, this study demonstrates for the first time that the bacterial siderophore pyoverdine (PVD) can directly alter the transcriptomic landscape of eukaryotic endothelial cells under sterile conditions, particularly affecting the expression of genes involved in iron metabolism. At the functional level, PVD exposure impairs vascular endothelial barrier integrity and reduces cell viability through multiple mechanisms, including mitochondrial dysfunction, oxidative stress, and disruption of tight junction proteins. These alterations collectively lead to increased endothelial permeability and barrier dysfunction. Future studies are needed to further elucidate the molecular mechanisms linking PVD-induced iron dysregulation to endothelial barrier injury and to determine whether therapeutic strategies targeting iron metabolism or oxidative stress can mitigate PVD-induced endothelial damage. A deeper understanding of these mechanisms may reveal novel therapeutic targets and provide potential strategies for the prevention and treatment of diabetic vascular complications.

## Data Availability

The datasets presented in this study can be found in online repositories. The names of the repository/repositories and accession number(s) can be found in the article/supplementary material.
